# Characterising *SRD5A2* Gene Variants in 37 Indonesian Patients with 5-Alpha-Reductase Type 2 Deficiency

**DOI:** 10.1155/2019/7676341

**Published:** 2019-12-01

**Authors:** Nanis S. Marzuki, Firman P. Idris, Hannie D. Kartapradja, Alida R. Harahap, Jose R. L. Batubara

**Affiliations:** ^1^Eijkman Institute for Molecular Biology, Jakarta 10430, Indonesia; ^2^Doctoral Program in Medicine, Universitas Indonesia, Jakarta 10430, Indonesia; ^3^Department of Child Health, Universitas Indonesia, Jakarta 10430, Indonesia

## Abstract

The 5-alpha-reductase type 2 deficiency (5ARD2) is an autosomal recessive condition associated with impairment in the conversion of testosterone to dihydrotestosterone. This condition leads to undervirilisation in 46,XY individuals. To date, there have been more than 100 variations identified in the gene responsible for 5ARD2 development (steroid 5-alpha-reductase 2, *SRD5A2*). However, few studies have examined the molecular characterisation of Indonesian 5ARD2 cases. In the current study, we analysed 37 subjects diagnosed with 46,XY DSD (disorders of sex development) with confirmed variations in the *SRD5A2* gene. We examined results from testosterone/dihydrotestosterone (T/DHT) and urinary etiocholanolone/androsterone (Et/An) ratios, as well as from molecular and clinical analyses. Twelve variants in the *SRD5A2* gene were identified, and 6 of which were novel, namely, c.34–38delGinsCCAGC, p.Arg50His, p.Tyr136^*∗*^, p.Gly191Arg, p.Phe194Ile, and p.Ile253Val variants. Moreover, we determined that 20 individuals contained harmful mutations, while the remaining 17 variants were benign. Those containing harmful mutations exhibited more severe phenotypes with median external genitalia masculinisation scores (EMS) of 3 (1.5–9) and were more likely to be diagnosed at a later age, reared as female, and virilised at pubertal age. In addition, the respective sensitivities for detecting severe 5ARD2 cases using T/DHT (cutoff: 10) and urinary Et/An ratios (cutoff: 0.95) were 85% and 90%, whereas mild cases were only identified with 64.7% and 47.1% sensitivity, respectively. Although we were unable to identify clear correlations between genotypic and phenotypic characteristics in this study, we clearly showed that individuals who were homozygous or compound heterozygous for any of the harmful mutations were more likely to exhibit classic 5ARD2 phenotypes, lower EMS, female assignment at birth, and virilisation during puberty. These results serve to inform the development of improved clinical and molecular 5ARD2 diagnostic approaches, specifically in Indonesian patients.

## 1. Introduction

Steroid 5-alpha-reductase type 2 deficiency (5ARD2) is a rare autosomal recessive disorder, caused by failure of testosterone to convert to dihydrotestosterone (DHT), which is an androgen that is 10 times more biologically active than testosterone [1, 2]. During foetal development, DHT induces the development of male external genitalia. Thus, reduced expression of DHT results in undervirilisation in affected 46,XY infants and clinical presentation that includes the presence of a micropenis, hypospadia, ambiguous genitalia, or external genitalia that resembles that of a female. Hence, individuals presenting with the classic form of 5ARD2 are often raised as females, who then undergo masculinisation during puberty, resulting in female to male gender reassignment procedures being performed in 56–63% of these cases [3].

The T/DHT ratio is widely used in diagnostics for 46,XY disorder of sex development (DSD) cases suspected of 5ARD2. However, there is increasing evidence that this ratio may yield conflicting results [4–7]. A consensus has not been reached regarding the appropriate cutoff values required for diagnosis of 5ARD2 cases, and thus the T/DHT ratio is commonly reported to generate false negative results, most notably in prepubertal children. Alternatively, urinary steroid profiling (USP) for 5*β*/5*α* metabolites and confirmatory molecular analysis of *SRD5A2* gene is employed in the diagnosis of 5ARD2.

The 5-alpha-reductase type 2 enzyme is encoded by the *SRD5A2* gene, which is located on chromosome 2p23. More than 100 mutations throughout the coding and flanking intronic regions of *SRD5A2* have been identified as associated with 5ARD2 development (The Human Gene Mutation Database, http://www.hgmd.cf.ac.uk/ac/index.php, accessed on February 2019). Since the DHT test is unavailable routinely in Indonesia, due to low demand, and is routinely sent abroad for analysis, the alternative tests are important to be set up in the diagnosis of 5ARD2. Following our previous report that identified two novel mutations in two siblings diagnosed with 5ARD2 [8], few reports on the molecular characterisation of 5ARD2 cases in the Indonesia population exist. The current study, therefore, examines the molecular and clinical characteristics of 37 Indonesian individuals diagnosed with 5ARD2.

## 2. Materials and Methods

### 2.1. Participant Enrolment and Initial Assessments

All individuals enrolled in our study were previously diagnosed with 46,XY DSD and had been referred for chromosomal analysis between July 2016 and September 2018. Only those with *SRD5A2* gene variants identified in both alleles were included in the study. To ensure the mutations occurred in both alleles, molecular analysis of parents and siblings was also performed. The confirmed 5ARD2 subjects were further assessed, and their clinical and hormonal profiles were analysed, including the testosterone/dihydrotestosterone (T/DHT) and urinary etiocholanolone/androsterone (Et/An) ratios. The clinical appearance of external genitalia was also assessed using external genitalia masculinisation score (EMS) as previously described by Ahmed and Rodie [9].

Informed consent was obtained from all participants as well as from all relevant family members. The study was approved by the ethical committee at the Faculty of Medicine, Universitas Indonesia.

### 2.2. Molecular Analysis of *SRD5A2* Genes

Genomic DNA was isolated from peripheral blood leucocytes using salting out procedure as previously described by Miller et al. [10]. The exons 1–5 and flanking regions of *SRD5A2* gene were amplified via PCR using primers as described previously by Nie et al. [11]. However, we modified the exon 4 forward primer as follows: 5′-CCA AGA GGA TTC CAC CAA ACT C-3′. Each 25 *µ*L PCR reaction contained 10 pmol of forward and reverse primers, 5 *µ*L of 5x MyTaq Red PCR Buffer (Bioline, United Kingdom), 1 unit of MyTaq™ HS Red DNA Polymerase enzyme, 100 ng of DNA template, and double distilled water to account for the remaining volume. PCR conditions were as follows: 95°C initial denaturation for 5 minutes followed by 35 cycles of denaturation (95°C for 30 seconds), annealing for 40 seconds (exon 1: 71°C, exon 2: 52°C, exons 3 and 4: 64°C, and exon 5: 60°C), and elongation for 40 seconds at 72°C. Final elongation was performed for 5 minutes at 72°C. The resulting amplified PCR products were then sequenced using the ABI3730XL DNA Sequencer (Applied Biosystem, CA) and analysed with published reference sequences (GenBank: NG_008365.1).

### 2.3. Predicting the Effects of *SRD5A2* Gene Mutations

The effects of amino acid substitutions on the protein functions were studied *in silico* using the PROVEAN and POLYPHEN web software programs. The sequences of reference protein were obtained from the UniProt/Swiss-Prot database (P31213.1). Intronic variations were analysed using the web-based software program, Human Splicing Finder version 3.1, to determine whether the substitutions interfere with splice sites.

### 2.4. Hormonal Assays

Human chorionic gonadotropin (hCG) test was performed in prepubertal subjects with unpalpable testis (5 cases). In brief, 1500 IU of hCG was administrated subcutaneously for 3 consecutive days. Blood and urinary samples were collected prior to administering the first injection as well as 24 hours after the third injection. The highest values observed, in the basal sample and poststimulation samples, for T/DHT and urinary Et/An were used in further analysis. Testosterone levels were quantified using automated enzyme-linked immunosorbent assays, according to manufacturer's instructions (ELISA, TOSOH AIA-900), while DHT levels were measured using DHT-optimised ELISA (EIA-5761) and analysed by DRG Instruments GmbH (Germany).

Urinary Et and An quantification was performed as follows: urine was extracted using Waters Sep-Pak® (Milford, USA) cartridge columns, hydrolysed by the *Escherichia coli* (*E. coli*) enzyme, glucuronidase, reextracted on Sep-Pak cartridges, and derivatised using trimethylsilylation (MSTFA/NH_4_I/ethanethiol). The products were then analysed using a gas chromatographer (model 7890, Agilent Technologies) with an HP Ultra 1 capillary column (17 m × 0.20 mm I.D × 0.11 m film thickness), coupled to a mass spectrometer (model 5975, Agilent Technologies). The oven temperature was programmed with the following parameters: initial temperature of 180°C was held for 1 second and raised at rate of 3°C/min to 234°C, where it was held for 2 minutes, and then increased at a rate of 20°C/min to 280°C, where it was held for 5 minutes. Finally, the temperature was increased at a rate of 30°C/min to 310°C, and was held for 1 second.

## 3. Results

### 3.1. Molecular Characteristics

**Figure 1:**
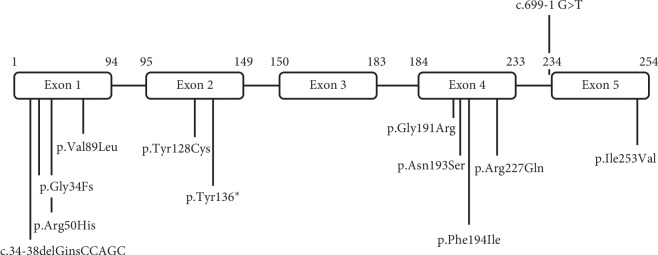
Distribution of the *SRD5A2* gene variants identified in 37 cases of 48,XY DSD. Most of the variants are located at exons 1 and 4 of the *SRD5A2* gene.

Thirty-seven 46,XY DSD cases were confirmed to have molecular defects in the *SRD5A2* gene. Following genomic analysis, 12 variations in the *SRD5A2* gene were detected, and 6 of which were identified as novel (Table 1) and many of which were identified in exon 1 (59/81) and exon 4 (11/81) (Figure 1). Moreover, two of the frameshift mutations detected in this study, specifically, the p.Gly34Fs and c.34–38delGinsCCAGC variants, had not been previously reported in other populations. These mutations were predicted to generate truncated proteins. The p.Gly34Fs mutation, which is caused by deletion of a guanine at codon 34, encodes a shortened protein composed of 40 amino acids; while the c.34–38delGinsCCAGC mutation causes the formation of a stop codon at sequence 136. Other novel mutations found in this study were all missense variants, including the p.ArgHis, p.Tyr136^*∗*^, p.Gly191Arg, p.Phe194Ile, and p.Ile253Val. Furthermore, the p.Val89Leu variant was the most frequent substitution detected and was identified in 43 of the 74 alleles (58.1%) that were examined.

### 3.2. Predicting the Effects of the *SRD5A2* Gene Mutations

Based on our *in silico* studies the p.Arg50His, p.Gly34Fs, p.Tyr128Cys, p.Gly191Arg, p.Asn193Ser, p.Phe194Ile, p.Tyr136^*∗*^, and p.Arg227Gln gene variants were determined to be harmful mutations; while the p.Val89Leu, and p.Ile253Val mutations were characterised as neutral. Furthermore, using web-based Human Splicing Finder software, the intronic mutation, c.699-1 G>T, which consists of a guanine being substituted with a thymine was determined to cause changes in splice sites. Additionally, the c.34–39delGinsCCAGC variant was characterised as deleterious because it is a frameshift mutation that caused truncated proteins to be encoded. According to the results from this genomic, we categorised the participants into two groups. The first group consisted of 20 individuals who were identified as having harmful mutations and were classified as “severe” (Table 2). The second “mild” group contained 17 individuals identified as either homozygous or compound heterozygous for benign mutations (Table 3).

### 3.3. Genotype-Phenotype Correlations

There were 5 sets of siblings or twins enrolled in the study. We accidently found out the subjects, S068 and S069, to have 5ARD2 by performing family study of S004. S068 was the father of S004. Age at diagnosis ranged from 0–41.8 years. However, the subjects in the severe group were diagnosed at a later age (mean 15.6 ± 12.0 years) compared to those in the mild group who were diagnosed at a median age of 0.5 years (0.0–10.0 years). Furthermore, subjects in the severe group were more likely to be reared as female, whilst subjects in the mild group were most often raised as males. Thirteen out of 20 subjects carrying harmful mutations were raised as female, while all mild subjects were raised as male (Table 2). As they entered puberty all female-raised subjects underwent virilisation, and consequently all of them, save for one (S010), decided to change their gender identity to male. Furthermore, all 8 subjects who were identified as homozygous for harmful mutations were raised as female with severe undervirilisation of external genitalia and palpable gonads (EMS = 3). Moreover, 7 of these individuals were not diagnosed until after puberty.

In prepubertal subjects (22), the levels of T and DHT were 0.25 (0.01–12.72) ng/ml, and 1.89 (0.01–307.28) pg/ml, respectively, while in pubertal participants (15), the levels were 6.72 ± 3.28 ng/ml, and 387.60 ± 155.63 pg/ml, respectively. A high T/DHT ratio, of more than 10, was identified in 85% (17 out of 20) of the participants in the severe group and in 64.7% (11/17) of the individuals in the mild group. Thus, overall sensitivity of the T/DHT ratio for the diagnosis of 5ARD2 was determined to be 75.7%. Additionally, the use of the urinary Et/An ratio with a cutoff of 0.95 was identified in 18 of the 20 individuals with severe mutations (90%) and in 8 of the 17 with mild mutations (47.1%) (Tables 2 and 3).

**Figure 2:**
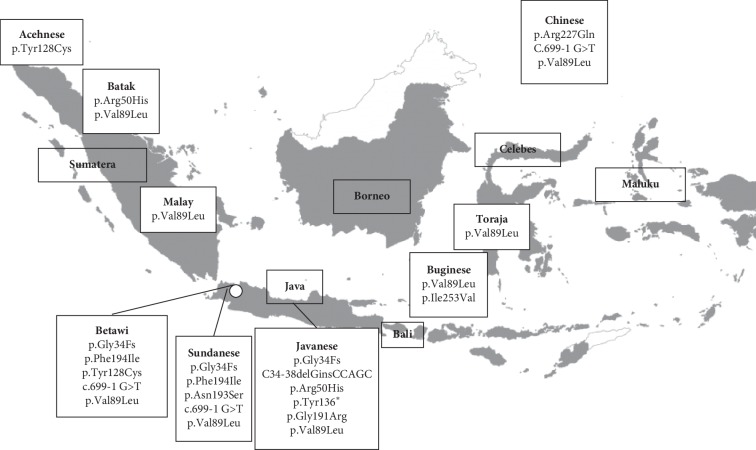
The geographical distribution of the identified *SRD5A2* gene variants as determined by the ancestral place of origin for each participant. Transparent boxes indicate major islands in Indonesia; white boxes indicate SRD5A2 gene variations based on subjects' ethnic origins. Acehnese, Batak, and Malay are originated from Sumatera island; the Sundanese, Betawi, and Javanese reside in Java island; and Buginese and Toraja are from South Celebes. The Indonesian Chinese people reside in almost all of Indonesian islands, especially in the major islands. White dot (◦) indicates Jakarta, the capital city of Indonesia.

All of the 5ARD2 subjects in this study ethnically originated from Indonesia, save for 4 cases, who were identified as Indonesian Chinese (S004, S017, S068, and S069 (Table 2)). The geographical distribution of the participants' ethnic heritage is depicted in Figure 2, which subsequently demonstrates the origin of the *SRD5A2* gene variants present in each individual.

## 4. Discussion

Diagnosis of 5ARD2 is suspected when individuals with 46,XY present with undervirilisation of external genitalia and increased T/DHT ratios. However, molecular analysis of *SRD5A2* gene variants is generally required to confirm the diagnosis, since the hormonal and clinical profiles of 5ARD2 often overlap with other conditions, including androgen insensitivity syndrome, 17 beta-hydroxylase deficiency and defects in the *NR5A1* gene.

Herein, we reported 37 46,XY DSD individuals with 5ARD2. Molecular genomic analysis of these individuals revealed 12 variants in the *SRD5A2* gene, and 6 of which had never been previously reported. Two siblings that had been previously reported in our earlier study [8] bearing the p.34GlyFs and c.699-1 G>T mutations were included in this study and were identified as S001 and S002. Moreover, the most frequently identified deleterious substitution detected in this series was the p.34GlyFs, which was carried by 8 participants (12 alleles). This mutation is caused by guanine deletion at codon 34 of the *SRD5A2* gene and is predicted to cause the production of a truncated 40-amino-acid protein. The 12 subjects identified as carriers of this mutation, in either the homozygous or heterozygous form, all presented with severe phenotypes resulting in their being reared as females and being assigned a median EMS of 3. Moreover, the previously reported missense mutations, p.Gly34Arg and p.Gly34Trp, occur in the same codon and led to variable clinical manifestations. Specifically, an Italian newborn carrying both the Gly34Trp substitution mutation and the p.Ala49Thr variant presented with a micropenis, a relative mild phenotype. The latter variant has been shown to increase the 5-alpha-reductase type 2 enzymatic activity [12] and might contribute in the boy's phenotype. In addition, homozygosity for the p.Gly34Arg substitution was characterised only in individuals of Egyptian heritage [13] and was reported to cause severe phenotypes with median EMSs of 3.33 [14]. All of these mutations are located in exon 1 of the *SRD5A2* gene and are known to affect the binding affinity (km) of testosterone. Thus, the phenotypic severity is associated with the specific location of the mutations and with trans-allelic variations in the gene [12, 14].

A further 9 of the identified *SRD5A2* variants were missense mutations, which are common forms of mutation identified within the *SRD5A2* gene (HGMD, accessed February 2019), which mostly involves missense mutations (74 out of 106). Furthermore, among the 6 novel variants that were identified, a small indel mutation, c.34–38delGinsCCAGC, was characterised in exon 1 and was predicted to shorten the encoded protein to 136 amino acids. This indel mutation was identified in a 14-year-old girl (S003), who experienced virilisation as she entered puberty. She concurrently carried the p.34GlyFs mutation, which resulted in a severe phenotype and an EMS of 3. This is the 4^th^ report that has identified small indel mutations in the *SRD5A2* gene. Previously, small indel mutations were characterised in Turkish [15], Chinese [11], and Mexican [16] 5ARD2 cases, all of whom presented clinically with either female or ambiguous external genitalia. While the location and number of bases involved in these indel mutations were variable, the resulting clinical manifestations were consistently severe.

The p.Phe194Ile variant was the second most commonly identified *SRD5A2* missense mutation in this study. Two Sundanese siblings (S009 and S010) presenting with an EMS of 3 were identified as homozygous for this mutation. Both of these individuals were raised as females, and subsequent virilisation occurred as they entered puberty. Another subject (S004) bearing this variant also contained the splice site mutation, c.699-1 G>T, while also being homozygous for the p.Val89Leu mutation. This individual exhibited moderate clinical presentations with an EMS of 5 and was raised as male. An additional two participants (S068 and S069) who were carriers of this mutation and the p.Arg227Gln and p.Val89Leu variants, also exhibited milder clinical presentations with an EMS of 8 and were even capable of parenting offspring. The broad spectrum of clinical presentation associated with 5ARD2 cases may be explained by varying levels of residual enzyme activity allowing for different levels of binding to androgen receptors, and 5-alpha-reductase type 1 activity [17–19]. Although we did not perform *in vitro* or *in vivo* studies for the mutations detected in this study, we have clearly demonstrated that c.699-1 G>T substitutions lead to milder phenotypes.

The remaining novel mutations identified in this study were the p.Tyr131^*∗*^ and p.Gly191Arg variants that were carried by a 16-year-old Javanese subject (S058), who was reared as female and experienced virilisation as she entered puberty. She presented with a severe phenotype and an EMS of 3. She also had increased T/DHT and urinary Et/An ratios. The substitution of cytosine with adenine at codon 131 has been shown to generate a stop codon, while the p.Gly191Arg variant may interfere with the binding affinity of 5-alpha-reductase for its cofactor, NADPH.

Some of the variants that we identified in our analysis, such as the p.Tyr128Cys, p.Asn193Ser, and p.Arg227Gln substitutions have been reported previously. The first identified case of the *SRD5A2* p.Tyr128Cys substitution was characterised in a 2-year-old Thai individual, who was reared as female and carried this mutation in combination with the p.Arg227Gln variant [20]. Thus, to our knowledge, our report is the second to describe this substitution, which, in our study, was identified in its homozygous form in two female-reared Acehnese siblings who presented with an EMS of 3, and an increased ratio of urinary Et/An. This mutation was predicted to have damaging effect on the protein and was located within a highly conserved sequence of the *SRD5A2* gene [20]. The p.Asn193Ser variant has also been identified in 5ARD2 cases originating from Thailand [20] and Spain [21]. In our study, however, a Sundanese 27-old female was found to carry this mutation in combination with the p.Gly34Fs variant. She made the decision to officially change her gender identity to male after experiencing virilisation during puberty and beginning a relationship with a female. The p.Arg227Gln substitution, which has also been described in several cases originating from Asian countries [6, 20, 22–24], was detected in 3 Chinese subjects (4 alleles) in our study. As previously mentioned, the 5ARD2 subjects carrying homozygous or heterozygous p.Arg227Gln variants showed milder phenotypes with EMS of 8 and 9, which is in concordance with a review by Avendaño et al. [14] that described moderate phenotypes and average EMS of 8 as associated with homozygous p.Arg227Gln individuals. Furthermore, Makridakis et al. [12] reported that the p.Arg227Gln variant was associated with enzymatic activity that was reduced to 3.2% of normal level, allowing for partial virilisation of external genitalia to occur.

In the current study, the p.Va89Leu variant was the most commonly observed mutation in *SRD5A2*. This variant is considered to be a polymorphism, since it is also found in normal individuals. Nevertheless, Makridakis et al. [12] demonstrated that the substitution of valine with leucine at codon 89 may moderately decrease enzymatic activity by approximately 30%, and may be associated with the development of hypospadias in genetically male individuals [25]. We determined that the clinical presentation of subjects that are homozygous for p.Val89Leu were variable with EMS ranging from 0 to 10.5. It is possible that other genes are involved in the development of these phenotypes; however, since we did not perform molecular analysis on other genes involved in male sex determination, (*AR*, *17BHSD*, and *NR5A1*) we are unable to address this possibility, and as such, we are aware that this is a shortcoming of this study.

Several studies have also reported on the presence of compound heterozygosity of p.Val89Leu with other substitutions in the *SRD5A2* gene [21, 22, 26]. These cases all involved phenotypically male individuals with micropenis and hypospadias. In our study we identified 3 subjects with compound heterozygosity of p.Val89Leu and p.Arg50His, which has not been reported elsewhere. These patients were reared as males and had EMS of 0, 6, and 6. The p.Arg50His variation was located in exon 1 and may alter enzyme function by affecting the substrate (testosterone) binding or by reducing enzymatic activity similar to how variations at codon 49 (the p.Ala49Thr) and codon 51 (the Ala51Thr) have been described to alter enzymic functioning. Specifically, the Ala49Thr substitution substantially increased enzymatic activity by almost 5 times the normal level and has been described as potentially contributing to increased risk of prostate cancer in African-American and Hispanic men [12, 27]. Moreover, Ala51Thr moderately reduced enzyme activity by approximately 30% with similar effects to that observed in individuals with the p.Val89Leu variant [12].

In this study, a large proportion of the *SRD5A2* gene mutations were located in exon 1 (59/81, 72.8%) and exon 4 (11/81, 13.6%). These results agree with those of a study conducted by Maimoun et al. [26] that included 55 subjects from various ethnic and geographical origins. This study also identified that 35.8% and 21.75% of mutant alleles on the *SRD5A2* gene were located on exons 1 and 4, respectively. Studies from Turkey [15] and China [22] also revealed similar results. However, studies from Spain [21] and India [28] described mutations largely located on exons 2 and 4 and exons 1 and 5 of the *SRD5A2* gene, respectively. However, all reports, including ours, agree that *SRD5A2* mutations rarely occur on exon 3.

The measurement of steroid hormones using mass spectrometry-based methods has been reported to yield more reliable results compared with immunoassays, especially in detecting low concentrations, i.e., testosterone and DHT in infants or women [29–31]. However, the use of LC-MS/MS method is challenging and not readily available in the whole regions; therefore, the appropriate use of immunoassay-based methods is still supported for diagnosis and monitoring of DSD [32]. Similarly, in our country, the immunoassay-based methods were routinely performed for testosterone measurements.

The immunometric assays are substantially accurate in the steroid range of pubertal participants, whose serum T is above 1 ng/ml. On the contrary, the accuracy of these assays in the prepubertal range fails in most of the cases and consequently limiting the interpretation of the T/DHT ratio. This main shortcoming of our study should be diminished by performing the hCG stimulation test. However, the hCG test is not reliably available in Indonesia and thus cannot be performed in all prepubertal individuals who require it. In our study, this was one of the limiting factors to performing hCG test on all prepubertal participants, even though it is known to yield higher sensitivity of the T/DHT ratio for diagnosis of 5ARD2 [30]. We observed that the T/DHT ratio corroborated with molecular analysis results only in those cases involving the presence of harmful mutation in both alleles. All of the “severe” participants in our study, save for one (S002), had T/DHT ratios greater than 10, and the overall sensitivity of this ratio was determined to be 75.7%, which was similar to that reported in another study [26], with a 72% sensitivity detected. Additionally, the T/DHT ratio exhibited stronger predictive capacity in postpubertal cases, most notably in those with severe mutations. We, therefore, believe that confirmation testing involving molecular genomic analysis of *SRD5A2* mutations would substantially improve the current diagnostic protocols for 5ARD2 cases in Indonesia. However, this diagnostic tool is currently only available in Jakarta and is considered to be expensive.

Urinary steroid profiling using the ratio of urinary steroid 5*β*/5*α* metabolites, including the ratio of etiocholanolone/androsterone, tetrahydrocorticosterone/5*α*-tetrahydrocorticosterone (THB/5*α*THB), and tetrohydrocortisol/5*α*-tetrahydrocortisol (THF/5*α*THF), is applied alternatively in the diagnosis of 5ARD2. We performed urinary Et/An ratio analysis in this study since it was the only urinary steroid 5*β*/5*α* metabolite ratio available in Indonesia. We set the cutoff at 0.95 based on our concurrent study and found significant discrepancies in the detection rate compared with the use of normal reference values previously published by Chan et al. [6]. By employing a cutoff of 0.95, the Et/An urinary ratio agreed with the corresponding *SRD5A2* genetic analysis in 90% and 47.1% of severe and mild cases, respectively. However, when the reference value set by Chan et al. [6] was employed, the results were in agreement in only 75% and 17.6% of cases, respectively. It has been suggested that an effective and sensitive means for diagnosing 46,XY DSD cases would include screening *SRD5A2* gene mutations in cases with urinary Et/An ratios higher than 1.49 (or an An/Et ratio less than 0.67) [33], a much higher cutoff than our finding and therefore should lower the detection rate when applied to our series. Variations in the normal reference values exist between research groups likely due to the differences in ethnic/genetic backgrounds of the participants and in the specific methodologies employed for each assay. Nevertheless, within our study, the urinary-based ratio demonstrated better detection sensitivity than did the T/DHT ratio, specifically in severe cases.

Since the first reports of 5ARD2 cases in the Dominican Republic [2] and the United States [34], many people have been diagnosed with this condition globally. Some studies have reported 5ARD2 originating from clustered or inbred cases in the same geographically isolated areas [2, 35–37] or from specific ethnic backgrounds [22, 28, 38–40]. This report, however, describes 5ARD2 cases from various ethnic backgrounds in Indonesia. The study was not a population-based study but rather enrolled participants based on those presenting to our clinic. Indonesia is a populous country, with more than 300 ethnic groups inhabiting more than 900 islands. The ethnic backgrounds of the subjects in this study were representative of the major ethnic groups in Indonesia. Our clinic is located in Jakarta, and therefore the referral cases mostly originated from the western region of Indonesia, specifically from Java (20/37 cases). The p.Val89Leu substitution variant was identified in nearly all the ethnic groups, while the patterns for the other mutations were much more irregular. Specifically, the Javanese, Sundanese, and Betawi subjects residing on the island of Java carried different mutations than individuals from the Sumatera and Celebes islands. The p.Gly34Fs variant was detected only in subjects from Java island, and thus whether specific *SRD5A2* mutations are associated with different ethnic groups in Indonesia is an area that warrants further investigation.

## 5. Conclusions

Twelve variants of the *SRD5A2* gene were identified in association with 5ARD2. Six of these mutations were novel and had not been previously reported. Although no distinct relationships were observed between genotypic and phenotypic characteristics in the participants, we did clearly demonstrate that individuals that are homozygous or compound heterozygous for harmful mutations were more likely to exhibit classic phenotypes for 5ARD2, including lower EMS, female assignment at birth, and virilisation during puberty.

## Figures and Tables

**Table 1:** Variations of the *SRD5A2* genes detected in Indonesian cases of 5ARD2.

Variations	Base change	Subjects (*n* = 37)	Alleles (*n* = 74)
Point mutations			
p.Arg50His^#^	CGC–CAC	3	3
p.Val89Leu	GTA–CTA	26	43
p.Tyr128Cys	TAC–TGC	2	4
p.Tyr136^*∗*#^	TAC–TAA	1	1
p.Gly191Arg^#^	GGA–AGA	1	1
p.Asn193Ser	AAT–AGT	1	1
p.Phe194Ile^#^	TTC–ATC	3	5
p.Arg227Gln	CGA–CAA	3	4
p.Ile253Val^#^	ATC–GTC	1	1
Frameshift mutations			
c.34–38delGinsCCAGC^#^	CTGGCAGGC–CTGCCAGCCAGGC	1	1
p.Gly34FsX40	TACGGGAAG–TACGGAAG	8	12
Intronic mutation			
c.699-1 G>T	ttttagGTTC–ttttatGTTC	5	5
Total variations in alleles			81

^#^New mutations.

**Table 2:** Results from molecular, clinical, and hormonal analysis of subjects carrying harmful *SRD5A2* mutations.

No.	Subject	Mutations	Ethnic background	Age at (years)		Sex rearing (M/F)	EMS	T (ng/ml)	DHT (pg/ml)	T/DHT ratio	Et/An ratio
				Diagnosis	Hormonal analysis						
1	S007	p.[Gly34Fs]; [Gly34Fs]	Sundanese	11.0	22.7	F	3	7.35	452.00	16.26	6.03
2	S008	p.[Gly34Fs]; [Gly34Fs]	Sundanese	17.0	33.9	F	3	4.48	372.00	12.04	11.55
3	S021	p.[Gly34Fs]; [Gly34Fs]	Javanese	13.0	21.3	F	3	4.88	394.00	12.39	6.45
4	S026	p.[Gly34Fs]; [Gly34Fs]	Javanese	26.0	27.3	F	3	1.97	188.00	10.48	4.05
5	S009	p.[Phe194Ile]; [Phe194Ile]	Sundanese	18.0	21.6	F	3	11.45	490.00	23.37	4.31
6	S010	p.[Phe194Ile]; [Phe194Ile]	Sundanese	13.0	16.8	F	3	9.26	304.00	30.46	3.5
7	S064	p.[Tyr128Cys]; [Tyr128Cys]	Acehnese	5.0	6.4	F	3	0.11	0.01	11,000.00	3.76
8	S065	p.[Tyr128Cys]; [Tyr128Cys]	Acehnese	23.5	23.5	F	3	10.27	376.10	3.93	4.03
9	S001	p.[Gly34Fs]; [c.699-1G>T; p.Val89Leu]	Betawi	18.0	26.3	F	3	6.41	357.95	17.91	8.32
10	S002	p.[Gly34F]; [c.699-1G>T; p.Val89Leu]	Betawi	14.0	22.2	F	3	6.71	702.00	9.56	12.23
11	S027	p.[Gly34Fs]; [Asn193Ser]	Sundanese	27.0	27.4	F	3	10.85	618.00	17.56	9.54
12	S003	p.[Gly34Fs]; c.[34-38delGinsCCAGC]	Javanese	14.0	14.1	F	3	6.47	240.00	26.96	3.44
13	S012	p.[Arg50His]; [Val89Leu]	Javanese	0.5	5.1	M	0	0.01	2.00	5.00	1.20
14	S015	p.[Arg50His]; [Val89Leu]	Batak	7.0	8.9	M	6	0.85	39.00	21.79	0.51
15	S019	p.[Arg50His; Val89Leu]; [Val89Leu]	Javanese/Batak	1.7	1.7	M	6	0.38	23.00	16.52	0.44
16	S004	[c.699-1G>T; p.Val89Leu]; p.[Phe194Ile; Val89Leu]	Sundanese/Chinese	0.4	5.3	M	6	0.74	12.00	61.67	4.32
17	S017	p.[Arg227Gln; Val89Leu]; [Arg227Gln]	Chinese	2.8	2.8	M	9	0.69	1.00	690.00	5.16
18	S068	p.[Arg227Gln]; [c.699-1G>T; p.Val89Leu]	Sundanese/Chinese	41.8	41.8	M	8	8.72	321.53	27.12	2.68
19	S069	p.[Arg227Gln]; [c.699-1G>T; p.Val89Leu]	Sundanese/Chinese	41.8	41.8	M	8	8.30	230.56	36.00	3.39
20	S058	p.[Tyr136^*∗*^]; [Gly191Arg]	Javanese	16.3	16.3	F	1.5	3.67	192.14	19.10	2.99

An: androsterone; DHT: dihydrotestosterone; EMS: external genitalia masculinization score; Et: etiocholanolone; F: female; M: male; T: testosterone. S001 and S002; S007 and S008; S009 and S010; S064 and S065 were siblings; S068 and S069 were twins; S004 was the son of S068.

**Table 3:** Results from molecular, clinical, and hormonal analysis of subjects carrying benign mutations in both alleles.

No.	Subject	Mutations	Ethnic background	Age at (years)		Sex rearing M/F	EMS	T (ng/ml)	DHT (pg/ml)	T/DHT ratio	Et/An ratio
				Diagnosis	Hormonal analysis						
1	S056	p.[Val89Leu]; [Ile253Val]	Buginese	0.3	2.4	M	9.5	0.03	0.08	375.00	1.03
2	S016	p.[Val89Leu]; [Val89Leu]	Batak	5.0	5.1	M	8.0	0.14	20.00	7.00	1.17
3	S020	p.[Val89Leu]; [Val89Leu]	Batak	10.0	11.4	M	3.0	0.37	65.00	5.69	0.73
4	S025	p.[Val89Leu]; [Val89Leu]	Batak	6.0	6.0	M	3.0	0.08	0.001	80.00	0.53
5	S034	p.[Val89Leu]; [Val89Leu]	Toraja	0.2	1.9	M	3.0	0.36	0.32	1,125.00	1.03
6	S035	p.[Val89Leu]; [Val89Leu]	Javanese	9.0	14.0	M	10.5	12.72	307.28	41.40	0.36
7	S037	p.[Val89Leu]; [Val89Leu]	Javanese	0	7.6	M	1.5	0.13	22.81	5.70	2.37
8	S040	p.[Val89Leu]; [Val89Leu]	Javanese	0.1	5.5	M	0	0.01	0.10	100.00	1.15
9	S041	p.[Val89Leu]; [Val89Leu]	Javanese	0	7.7	M	0	0.04	0.03	1,333.33	0.03
10	S042	p.[Val89Leu]; [Val89Leu]	Javanese	6.0	6.8	M	9.0	0.03	281.25	0.11	0.55
11	S047	p.[Val89Leu]; [Val89Leu]	Malay	0.4	0.5	M	6.0	1.92	1.77	1,084.75	0.35
12	S048	p.[Val89Leu]; [Val89Leu]	Betawi	0.1	0.1	M	6.0	1.13	132.51	8.53	0.24
13	S050	p.[Val89Leu]; [Val89Leu]	Javanese	1.5	1.5	M	3.0	0.05	0.44	113.64	0.51
14	S055	p.[Val89Leu]; [Val89Leu]	Batak	1.0	1.5	M	6.0	0.70	0.01	70,000.00	1.01
15	S063	p.[Val89Leu]; [Val89Leu]	Buginese	6.0	10.4	M	3.0	0.06	575.69	0.10	0.32
16	S066	p.[Val89Leu]; [Val89Leu]	Batak	0.4	0.4	M	3.0	0.65	14.62	44.46	3.58
17	S067	p.[Val89Leu]; [Val89Leu]	Javanese	0.5	2.4	M	2.0	0.08	1.07	74.77	4.20

An: androsterone; DHT: dihydrotestosterone; EMS: external genitalia masculinization score; Et: etiocholanolone; F: female; M: male; T: testosterone.

## Data Availability

The clinical, ethnic, hormonal, and molecular data used to support the findings of this study are included within the article.

## References

[1] Wilson J. D., Griffin J. E., Russell D. W. (1993). Steroid 5 alpha-reductase 2 deficiency. *Endocrine Reviews*.

[2] Imperato-McGinley J., Guerrero L., Gautier T., Peterson R. E. (1974). Steroid 5agr-reductase deficiency in man: an inherited form of male pseudohermaphroditism. *Science*.

[3] Cohen-Kettenis P. T. (2005). Gender change in 46,XY persons with 5*α*-reductase-2 deficiency and 17*β*-hydroxysteroid dehydrogenase-3 deficiency. *Archives of Sexual Behavior*.

[4] Walter K. N., Kienzle F. B., Frankenschmidt A. (2010). Difficulties in diagnosis and treatment of 5*α*-reductase type 2 deficiency in a newborn with 46,XY DSD. *Hormone Research in Paediatrics*.

[5] Bertelloni S., Scaramuzzo R. T., Parrini D., Baldinotti F., Tumini S., Ghirri P. (2007). Early diagnosis of 5*α*-reductase deficiency in newborns. *Sexual Development*.

[6] Chan A. O. K., But B. W. M., Lee C. Y. (2013). Diagnosis of 5-reductase 2 deficiency: is measurement of dihydrotestosterone essential?. *Clinical Chemistry*.

[7] Perry R. J., Novikova E., Wallace A. M., Donaldson M. D. C. (2011). Pitfalls in the diagnosis of 5*α*-reductase type 2 deficiency during early infancy. *Hormone Research in Paediatrics*.

[8] Marzuki N. S., Suciati L. P., Dewi M., Tridjaja B. (2010). Two novel mutations of SRD5A2 gene in Indonesian siblings with clinical 5-alpha-reductase deficiency. *Journal of Pediatric Endocrinology and Metabolism*.

[9] Ahmed S. F., Rodie M. (2010). Investigation and initial management of ambiguous genitalia. *Best Practice & Research Clinical Endocrinology & Metabolism*.

[10] Miller S., Dykes D., Polesky H. (1988). A simple salting out procedure for extracting DNA from human nucleated cells. *Nucleic Acids Research*.

[11] Nie M., Zhou Q., Mao J., Lu S., Wu X. (2011). Five novel mutations of SRD5A2 found in eight Chinese patients with 46,XY disorders of sex development. *Molecular Human Reproduction*.

[12] Makridakis N. M., Di Salle E., Reichardt J. K. V. (2000). Biochemical and pharmacogenetic dissection of human steroid 5?-Reductase type II. *Pharmacogenetics*.

[13] Gad Y. Z., Khairt R., Mazen I., Osman H. G. (2007). Detection of the G34R mutation in the 5 alpha reductase 2 gene by allele specific PCR and its linkage to the 89L allele among Egyptian cases. *Sexual Development*.

[14] Avendaño A., Paradisi I., Cammarata-scalisi F., Callea M. (2018). 5-*α*-Reductase type 2 deficiency: is there a genotype-phenotype correlation? A review. *Hormones*.

[15] Akcay T., Fernandez-cancio M., Turan S., Güran T., Audi L., Bereket A. (2014). ARandSRD5A2gene mutations in a series of 51 Turkish 46,XY DSD children with a clinical diagnosis of androgen insensitivity. *Andrology*.

[16] Vilchis F., Valdez E., Ramos L., García R., Gómez R., Chávez B. (2008). Novel compound heterozygous mutations in the SRD5A2 gene from 46,XY infants with ambiguous external genitalia. *Journal of Human Genetics*.

[17] Marchetti P. M., Barth J. H. (2013). Clinical biochemistry of dihydrotestosterone. *Annals of Clinical Biochemistry*.

[18] Thiele S., Hoppe U., Holterhus P.-M., Hiort O. (2005). Isoenzyme type 1 of 5alpha-reductase is abundantly transcribed in normal human genital skin fibroblasts and may play an important role in masculinization of 5alpha-reductase type 2 deficient males. *European Journal of Endocrinology*.

[19] Imperato-McGinley J., Peterson R. E., Gautier T. (1982). Hormonal evaluation of a large kindred with complete androgen insensitivity: evidence for secondary 5*α*-reductase deficiency. *The Journal of Clinical Endocrinology & Metabolism*.

[20] Ittiwut C., Pratuangdejkul J., Supornsilchai V. (2016). Novel mutations of the SRD5A2 and AR genes in Thai patients with 46,XY disorders of sex development. *Journal of Pediatric Endocrinology and Metabolism*.

[21] Fernández-Cancio M., Audí L., Andaluz P. (2011). SRD5A2 gene mutations and polymorphisms in Spanish 46,XY patients with a disorder of sex differentiation. *International Journal of Andrology*.

[22] Cheng J., Lin R., Zhang W. (2015). Phenotype and molecular characteristics in 45 Chinese children with 5*α*-reductase type 2 deficiency from South China. *Clinical Endocrinology*.

[23] Samtani R., Bajpai M., Ghosh P. K., Saraswathy K. N. (2015). A49T, R227Q and TA repeat polymorphism of steroid 5 alpha-reductase type II gene and Hypospadias risk in North Indian children. *Meta Gene*.

[24] Sasaki G., Ogata T., Ishii T. (2003). Micropenis and the 5*α*-reductase-2 (SRD5A2) gene: mutation and V89L polymorphism analysis in 81 Japanese patients. *The Journal of Clinical Endocrinology & Metabolism*.

[25] Thai H. T. T., Kalbasi M., Lagerstedt K., Frisén L., Kockum I., Nordenskjöld A. (2005). The valine allele of the V89L polymorphism in the 5-*α*-Reductase gene confers a reduced risk for hypospadias. *The Journal of Clinical Endocrinology & Metabolism*.

[26] Maimoun L., Philibert P., Cammas B. (2011). Phenotypical, biological, and molecular heterogeneity of 5*α*-reductase deficiency: an extensive international experience of 55 patients. *The Journal of Clinical Endocrinology & Metabolism*.

[27] Makridakis N. M., Ross R. K., Pike M. C. (1999). Association of mis-sense substitution in SRD5A2 gene with prostate cancer in African-American and Hispanic men in Los Angeles, USA. *The Lancet*.

[28] Shabir I., Khurana M. L., Joseph A. A., Eunice M., Mehta M., Ammini A. C. (2015). Phenotype, genotype and gender identity in a large cohort of patients from India with 5*α*-reductase 2 deficiency. *Andrology*.

[29] Kamrath C., Wudy S. A., Krone N., Hiort O., Ahmed S. F. (2014). Steroid biochemistry. *Endocrine Development*.

[30] Bertelloni S., Russo G., Baroncelli G. I. (2018). Human chorionic gonadotropin test: old uncertainties, new perspectives, and value in 46,XY disorders of sex development. *Sexual Development*.

[31] Taieb J. (2003). Testosterone measured by 10 immunoassays and by isotope-dilution gas chromatography-mass spectrometry in sera from 116 men, women, and children. *Clinical Chemistry*.

[32] Kulle A., Krone N., Holterhus P. M. (2017). Steroid hormone analysis in diagnosis and treatment of DSD: position paper of EU COST Action BM 1303 “DSDnet”. *European Journal of Endocrinology*.

[33] Berra M., Williams E. L., Muroni B. (2011). Recognition of 5*α*-reductase-2 deficiency in an adult female 46XY DSD clinic. *European Journal of Endocrinology*.

[34] Walsh P. C., Madden J. D., Harrod M. J., Goldstein J. L., MacDonald P. C., Wilson J. D. (1974). Familial incomplete male pseudohermaphroditism, type 2. *New England Journal of Medicine*.

[35] Thigpen A. E., Davis D. L., Gautier T., Imperato-McGinley J., Russell D. W. (1992). The molecular basis of steroid 5*α*-reductase deficiency in a large dominican kindred. *New England Journal of Medicine*.

[36] Imperato-McGinley J., Miller M., Wilson J. D., Peterson R. E., Shackleton C., Gajdusek D. C. (1991). A cluster of male pseudohermaphrodites with 5*α*-reductase deficiency in Papua New Guinea. *Clinical Endocrinology*.

[37] Hochberg Z., Chayen R., Reiss N. (1996). Clinical, biochemical, and genetic findings in a large pedigree of male and female patients with 5 alpha-reductase 2 deficiency. *The Journal of Clinical Endocrinology & Metabolism*.

[38] Akgun S., Ertel N. H., Imperato-McGinley J., Sayli B. S., Shackleton C. (1986). Familial male pseudohermaphroditism due to 5-alpha-reductase deficiency in a Turkish village. *The American Journal of Medicine*.

[39] Canto P., Vilchis F., Chávez B. (1997). Mutations of the 5*α*-reductase type 2 gene in eight Mexican patients from six different pedigrees with 5*α*-reductase-2 deficiency. *Clinical Endocrinology*.

[40] Nordenskjöld A., Ivarsson S.-A. (1998). Molecular characterization of 5*α*-reductase type 2 deficiency and fertility in a Swedish family 1. *The Journal of Clinical Endocrinology & Metabolism*.

